# Defining Autonomy in Psychiatry

**DOI:** 10.3389/fpsyt.2022.801415

**Published:** 2022-05-31

**Authors:** Jessy Bergamin, Judy Luigjes, Julian Kiverstein, Claudi L. Bockting, Damiaan Denys

**Affiliations:** Department of Psychiatry, Amsterdam UMC, Amsterdam, Netherlands

**Keywords:** autonomy, authenticity, competence, mental illness, self, identity

## Abstract

Mental illness undermines a patient's personal autonomy: the capacities of a person that enables them to live a meaningful life of their own making. So far there has been very little attention given to personal autonomy within psychiatry. This is unfortunate as personal autonomy is disturbed in different ways in psychiatric disorders, and understanding how autonomy is affected by mental illness is crucial for differential diagnosis and treatment, and also for understanding personal recovery. We will argue that disturbance of personal autonomy is related to patient's diminished quality of life and suffering that motivates seeking treatment. We hypothesize that (1) personal autonomy is generally reduced by mental illness but (2) the effects on autonomy are expressed differently according to the underlying psychopathology, and also vary according to the (3) context, and perspective of the individual patient. We provide a discussion of how autonomy can be affected in five prototypical mental disorders; Major Depressive Disorder, Substance-use Disorders, Obsessive Compulsive Disorder, Anorexia Nervosa and Schizophrenia. We take these disorders to be illustrative of how diminished autonomy is a central but overlooked dimension of mental illness. We will use our discussion of these disorders as the basis for identifying key dimensions of autonomy that could be relevant to innovate treatment of psychiatric disorders.

## Introduction

While psychiatry has been concerned with the impact of mental disorders on people's lives, much less attention has been given how mental illness interferes with their personal autonomy: the person's ability to live a meaningful life of their own making. The relation between autonomy and psychiatric disorders is complicated and not well known. In developmental psychology movement toward greater autonomy is considered a hallmark of optimal development and autonomy is seen as central to healthy psychological development and functioning (1). Mental illness impacts a person's autonomy in many different ways which we will explore in this paper. Historically, clinical literatures have described disturbances in autonomy as characteristic for many behavioral and mental disorders. In many mental disorders people's behaviors, emotions, thoughts are experienced as pressured, compelled, controlled or alternatively incontrollable (1). For instance, the craving in addiction that corrodes the patient's ability to achieve their long-term goals. Or the given how a patient with obsessive compulsive disorder (OCD) may spend eight hours a day on compulsive rituals, making it difficult for them to find and hold down a job. Another example is a depressed patient that lacks all motivation to envision a future for themselves. Mental illness can also affect autonomy in a general way, through how people relate to themselves and the world around them. For instance, rigid dysfunctional beliefs (such as I am worthless) common in mental disorders, may prevent patients even attempting to pursue goals they find worthwhile. On the other hand, personal autonomy could affect the course of treatment of mental illness positively. Patients who experience being able to live a life of their own making despite their mental illness may feel more capable of committing to treatment. They may also have greater hope for recovery which might alleviate suffering and contribute to a beneficial treatment outcome [see for instance Perkins et al. 2008 (2)].

Understanding the relationship between autonomy and mental illness is potentially valuable and underexplored for psychiatry. The disturbance of autonomy, for instance, could impact the risk of onset, the severity of symptoms, as well as the maintenance of mental disorders. Conversely, for individuals who experience more autonomy, it may lessen the burden of their illness. The interaction between autonomy and mental illness is likely to differ between individuals, and could also show distinct patterns for different disorders, a hypothesis we explore later in this paper. By looking at the distinct patterns of disturbance of autonomy in different mental disorders, we were able to distinguish dimensions of autonomy we suggest are relevant for assessing autonomy in psychiatry. More specifically, we hypothesize that autonomy is (i) generally reduced by mental illness; (ii) the effects on autonomy are expressed differently according to the underlying psychopathology, and (iii) these effects are dependent on the context and perspective of the individual patient.

### The Interaction of Autonomy and Mental Illness

We will take personal autonomy to be an acquired set of capacities that allow a person to act in ways they determine to be fulfilling or worthwhile. There is longstanding debate about how to define autonomy within the fields of legal, moral and political philosophy, and practical ethics (3–10). This debate indicates how difficult it is to reach consensus about a definition of the concept of autonomy in terms of a set of necessary and sufficient conditions. Part of the difficulty with the concept of autonomy is what we might call the “thickness” of the concept. It can be interpreted in many different ways because the concept has figured historically in a wide variety of different legal, ethical and political contexts (11). In this paper, we specifically focus on how the patient's experience of autonomy can be affected in mental illness. We propose a working definition of autonomy that serves this purpose but that also fits with the use of this term in the wider philosophical literature (12).

The argument of our paper is that mental illness interferes with the set of capacities required for autonomy. Based on the DSM definition, mental illness can be characterized in terms of two conditions:(i) unmanageable distress and (ii) impairment of functioning (13). We describe the distress experienced in mental illness as “unmanageable” in order to distinguish disorders from everyday distress a person can experience. Exactly when distress is unmanageable is a personal matter, that strongly depends on the person's social situation and what is expected of them (14). Each of these dimensions of mental illness (distress and impairment) can impact on a person's autonomy. Psychological distress and suffering directly disturb autonomy, while impairment of functioning can indirectly disturb autonomy (14). For example, a depressive patient may feel they are unable to work, due to a lack of energy and focus caused by their depression. Just as a person's autonomy can be interfered with from the outside when they are forced to do something against their will, so mental illness can also interfere with autonomy from inside of the person.

### Two Dimensions of Autonomy: Competence and Authenticity

We will distinguish two dimensions of autonomy that mental illness can undermine, which we will refer to as ‘competence' and ‘authenticity'. In many theories of autonomy these dimensions are the two main pillars used to explain the concept of autonomy (6, 7, 10, 12, 15–21). Christman, for example, writes:

“In the recent philosophical literature, autonomy has been conceived as potentially embodying a variety of conditions. Some of these requirements relate to the agent's ability to form desires and make them effective. Such conditions relate to cognitive and normative competence –rationality, self-control, absence of psychosis and other pathologies, and so on. In addition, some have argued that autonomy means not only being able to act effectively on one's desires but also that such desires, values, or other springs of action are truly the agent's own. These requirements relate to the authenticity of the agent's desires and values and often include the requirement of critical self-reflection on the factors relative to which the person is autonomous.” [(16), p.134].

We will use this conceptual distinction to provide a fine-grained analysis of how mental illness disrupts autonomy. We will see later however that, in practice, the disturbance of these two conditions will typically go together.

#### The Competence Condition

We use the term *competence* as an umbrella term to refer to the person's ability to form goals and intentions and to act effectively on them. This requires among other things being able to weigh up different options, select between them the option that is preferred, and plan for how to achieve one's intentions, preferences and goals once they have been formed. It requires self-control understood as the capacity for resisting temptations that might throw the agent of course, diverting from the course of action they have judged to be best given their goals.

Mental illness can interfere with a person's competence in multiple ways. It can do so at the stage of the person's forming intentions, preferences and goals. Think of the person with obsessive compulsive disorder that devotes the whole day to cleaning their kitchen. They may realize there is no point to their cleaning – the kitchen is already spotless – but feel they have no alternative but to continue. Mental illness can also disturb a person's capacity for self-control. A gambler may not want to bet on a horse race but find they are unable to resist the temptation to do so. Mental illness can also deprive a person of the ability to act on their intentions and achieve their goals because of apathy and low-energy, or lack of self-belief and self-esteem and (self) stigma.

#### The Authenticity Condition

We use the term *authenticity* to refer to how the self relates to the beliefs, desires, commitments and values that motivate them to act, and rationalize their decisions. We will refer to this broad class of mental states under the heading of ‘motivational states.' A person can reflect on each of their motivational states, and consider whether to accept or reject them. The motivational states that the person endorses qualify as *authentic*, while those states the person would not endorse upon reflection are said to be *inauthentic*.

We would note that ambivalence about one's preferences is quite consistent with acting authentically. Inner conflict is a normal part of daily life, and ambivalence, doubt, indecisiveness is often an appropriate response to one's situation and does not necessarily diminish one's autonomy. If two values that are central to a person conflict with one another, ambivalence is sometimes the only way to stay true to oneself (22). As an example of such ambivalence, consider Schechtman's (23) case of a woman who equally values her role as a mother and her role as a professional. The woman has to choose between attending an important dance recital of her daughter or an important work meeting. Both motivations make an equally valid claim on her and there is no straightforward way for her to bring them into coherence with each other. Considering the multiple life goals people juggle in modern society, this ability to sometimes be ambivalent does not weaken autonomy and may perhaps even bolster it.

Consider as an example of inauthentic motivational states, the states that cause compulsive behaviors. These are behaviors that feel as if they are externally forced upon the person rather than having their source in motivational states the person endorses. Addiction is a classic example in which a person may continue using an addictive substance even though they do not want to (9). The drive to seek and use the substance is one the person rejects. Such a motivation is not (or is no longer) an expression of how they want to live their life.

Authenticity requires a sense of what is important in one's life and thus can be disturbed in individuals whose outlook on oneself and one's life is lacking or conflicted. Think for instance of a patient with schizophrenia who, after going through a psychosis, feels apathy toward most things in her life. She no longer has a clear sense of who she is, or what motivates her and consequently spends most of her time watching television and sleeping.

Mental illness therefore can interfere with authenticity when behavior, thoughts and feelings are affected in such a way that it no longer seems to be an expression of the motivational states they identify with or when symptoms cause them to lose their sense of self.

Whether a person's motivational states are authentic is a complicated question that should be considered over a longer period of time in the person's life. This is particularly relevant for considering autonomy in mental illness where many disorders are characterized by periods in which people are overwhelmed by emotions. For instance, consider a person suffering from anorexia nervosa that has reached a morbidly dangerous body weight. Further suppose that the person is admitted to hospital and helped to recover. Now at the time of their admission to hospital we can suppose this person may have been fully committed to avoid gaining weight at all costs, including perhaps their own life. However, this same person might view her situation differently after a period of treatment, and slowly gaining weight. Treatment may afford her a broader perspective on what she wants for herself. Here, from the vantage point of their future self this drive to maintain a low body weight is one the person no longer (fully) endorses. The authenticity of a person's motivational states should not be assessed at an isolated instant in time but in relation to the self that is extended in time, or what is sometimes called the ‘narrative self' (23).

### Situating Autonomy in a Social Context and Individual Differences

The examples we have given of how mental illness can deprive a person of their autonomy have all focused on how mental illness can interfere with autonomy from the inside of the individual. However, it would be a mistake to conclude that autonomy is therefore a capacity of the individual taken in isolation from their context, i.e., their societal context. A person who is highly perfectionistic may, for example, view her perfectionistic tendencies as an important part of living a fulfilling life but her perfectionism may also make it difficult for her to hold down a job. An excessively narrow focus on her individual experience may suggest that adaptation of her perfectionism for her work may affect her autonomy since she has to compromise her perfectionistic values. Yet, taking a wider perspective on her situation may lead to a different conclusion: such a compromise may actually bolster her autonomy by opening up new career possibilities, improving her relationship with her coworkers, and her overall satisfaction in work and life.

Autonomy has to be viewed in a broader context of relationships and the person's societal perspective, as well as the individual's perspective on their life within this context. For one person making such a compromise, while a difficult choice, may have increased their possibilities for living a fulfilling life. For another person, such a compromise, conscious or unconscious, may feel inauthentic contributing to distress and diminishing their ability to live the life they would like to live.

There are also likely to be individual differences in how one's autonomy is affected by social circumstances that limit a person's autonomy (22). An example is a woman who dreams about studying medicine and becoming a doctor, but lives in a society where she is expected to become a housewife. She may adopt new life goals of caring for her family that may give her a higher sense of autonomy compared to holding on to her unattainable dreams. In another person such a compromise could however lead to feelings of depression and emptiness because she experiences the social expectations of her as in conflict with the life she desires for herself. In another person this could lead to an improvement in autonomy by experiencing being able to adapt values that are attainable within ones circumstances. These inter- and intra-individual differences in perceptions of being autonomous exist and need to be seen in a wider perspective of the social and societal context and the perspective of that individual on life within their current life stage.

The person's belief in their own capabilities [referred to as ‘self-efficacy' (24)], is also dependent on the interplay between environmental circumstances and personal characteristics. Bound up with competence are beliefs of self-worth and esteem, self-respect and self-trust (25). Traumatic events, neglect, poverty, unstable or dangerous environment, learning disabilities can all negatively impact self-efficacy. Social experiences can lead one to conclude that there is no point to one's undertakings, and what one aspires to achieve is no longer worth pursuing (25).

Summing up our background discussion, we have distinguished between two ways in which mental illness can limit autonomy. Mental illness can affect autonomy through the disruption of competence and/or authenticity. Second, we have argued that the impact of mental illness on autonomy can vary across individuals in ways that depend strongly on the individual's social and societal context, one's personal history and one's perspective on life and their values at that moment in life. A longer period of time in the patient's life has to be taken into account when looking at the impact of mental illness on their autonomy. We should therefore expect significant individual differences in how much autonomy a person loses and in what way autonomy is affected as a consequence of a person's mental illness.

## Autonomy Subverting Effects of Mental Disorders

In this section we will explore the different ways in which autonomy can be affected in five mental disorders: Major Depressive Disorder (MDD), Substance Use Disorders (SUDs), Obsessive-Compulsive Disorder (OCD), Anorexia Nervosa and Schizophrenia. We base our classifications of disorders on DSM-V. We chose the DSM-V to define mental disorders because it is the most widely, commonly used diagnostic handbook in psychiatry and will therefore be comprehendible to the readers of our article. We base our argument on our own clinical observations, theoretical reasoning and relevant literature within the field. We will see how in these different mental disorders, competency and authenticity can be affected in different but overlapping ways.

### Autonomy in Major Depressive Disorder

Major Depressive Disorder (MDD) is defined as the presence of a depressed mood and/or a substantial decrease of interests or pleasure, accompanied by at least five of the following symptoms: significant weight loss or -gain, insomnia or hypersomnia, psychomotor retardation or agitation, loss of energy, feelings of worthlessness or guilt, loss of concentration or decision-making and recurrent suicidal thoughts and/or attempts. All of these symptoms have a profound impact on a patient's life (26). A patient suffering from MDD can experience a loss of energy, despair, hopelessness and a lack of direction in life resulting from a loss of interest. Many depressed patients experience difficulty in maintaining a daily structure and decrease of interest and engagement in activities or employment (14).

Failure in motivation and engagement in depression hampers autonomy in at least the following two ways. First, a depressed person still has values and desires relating to how they want to live, but they are not motivated to act upon them (27). This can happen because the agent becomes alienated from cultural or other external pressures that previously motivated them. However, as Calhoun (27) argues, in depression a person can also lose interest in their values and desires in the absence of these reasons. For some patients this may be related to the inability to foresee a future for themselves. Getting through the day can become a burden in itself leading to feelings of hopelessness. Also, MDD is associated with impaired cognitive functioning, which is not limited to the acute phase but persists when MDD has remitted (28). This impairment in cognitive functioning can compromise autonomy on a basic level. Impaired cognitive functioning associated with MDD is linked to reduced daily functioning (28). Depending on the severity of the depression and impairment in cognitive functioning, which can vary over time, this inability to envision a future or any future improvement in functioning and life, can lead to a despair, defeat and the feeling of entrapment. Entrapment without, among other factors, the belief in one's ability to form and reach goals, can lead, and to suicidal ideation and suicidal behavior (29). We hypothesize that in this motivational phase of suicidal ideation, competence (loss of belief in their ability to form and pursue goals) and authenticity (loss of interest in their values or absence of these values) are severely compromised. In addition, the recurrent suicidal thoughts common in this phase can become intrusive thinking and intrusive imagery, processes which are often reported in this pre-suicidal process (period in which a person gradually gravitates toward the act) (30). These intrusive thoughts and imagines can be experienced as forced upon the patient without any ability to control these processes, compromising competence and authenticity. However, in some patients the volition phase, in which a person plans the suicidal act, can increase the feeling of competence (29). With the suicidal plan, the patient may feel more in control over their circumstances by the ability to end one's suffering. Authenticity however, might still be compromised if the person ultimately desires to live without suffering rather than ending their life. This indicates that for some people the feeling of competence may change during the different phases of suicide planning, which may be an important factor for some people driving their suicidal ideation.

Another important threat to autonomy that often accompanies major depression, and which may also underlie the patient's lack of motivation and engagement, comes from what is sometimes called ‘learned helplessness' – the belief that one cannot significantly affect one's circumstances, and there is little point in trying (31). This can be related to often reported worthlessness, loss of concentration or decision-making and recurrent suicidal thoughts. When a patient experiences a loss of energy and has no ability to concentrate, the ability to make their intentions and plans effective is naturally reduced. This can also lead to feelings of worthlessness or inappropriate guilt that negatively impact self-confidence and the person's ability to achieve what is important to themselves.

It is also important to consider how the situation external to the person can limit the competence of the patient diagnosed with MDD. Trauma, poverty, discrimination, lack of psychological and physical safety may greatsly impact a person's belief in their ability to form, pursue and reach goals. Misfortune and an unsafe and changing environment, may lead one to abandon the whole pursuit of goals in the first place (27).

Making use of our analysis of competence and authenticity from section Introduction, we suggest that MDD affects autonomy in the following ways. It impacts competence in two respects: the person's lack of motivation has the consequence that they are unable to act to bring about intentions, goals and plans, even if these are intentions, goals and plans to which they remain committed. Second, their general lack of self-worth in combination with poor environmental factors may manifest as a loss of self-efficacy, and a learned helplessness. Authenticity is impacted in that they are estranged or alienated from their values and desires. The lack of motivation they experience can be described in terms of this estrangement – their values and desires cease to mean anything to them. Or they may lose interest in values and desires all together and be unable to envision a future for themselves. Furthermore, suicidal ideation and suicidal behavior can be present compromising competence and authenticity differently depending on the severity of the depressive symptoms and according to the phases of suicidal behavior a person engages in.

#### Autonomy in Substance Use Disorder

Substance use disorders (SUDs; our focus in this section) are defined as severe problems related to compulsive and repetitive, habitual use of a substance. It is characterized by at least two of the following features that occur within the period of a year: a persistent desire or unsuccessful effort to cut down or control the use of the substance. Craving, or a strong desire or urge to use the substance. A great deal of time is spent in activities necessary to obtain the substance, use the substance, or recover from its effects. Important social, occupational, or recreational activities are given up or reduced as a consequence of the use of the substance. The recurrent use of the substance often results in a failure to fulfill major role obligations at work, school, or home, while the use of the substance continues despite having persistent or recurrent social or interpersonal problems caused or exacerbated by the effects of its use. The use of the substance is recurrent in situations in which it is physically hazardous, and the use of the substance is continued despite knowledge of having a persistent or recurrent physical or psychological problem that is likely to have been caused or exacerbated by the substance (26).

A view of SUDs, which is still widely held, is that dependence destroys one's competence and patients cannot help themselves (32, 33). In this way, SUDs are often seen as involving a loss of self-control implying that people suffering from SUDs either have control over their behavior or they do not (34). Dependence, abuse and craving are often taken as prototypical examples of a person being governed by overwhelming desires and urges instead of acting based on authentic motivational states that cohere with other of their reasons and motivations (9). Yet a closer look at agency in SUDs paints a more complicated picture.

Substance use disorder related behavior can be characterized by a loss of self-control but also by choices made by the person with a SUD (35). Thus, these behaviors are in some sense intentional behaviors. To capture this intentionality, we will follow Snoek (34) in recognizing different levels of self-control that can be hierarchically ordered. For example, self-control at lower levels of the hierarchy consists of abilities for resisting temptations and remaining resolved, setting and sticking to short-term goals and intentions. Self-control at the highest level of the hierarchy is the ability to act authentically according to one's values, (34) while the lower levels of self-control are arguably crucial for competence. Studies have shown that addiction leads to changes in attentional and reward processing or a propensity to engage in habitual behaviors over goal directed behaviors (36–38). Yet, understanding addiction merely as a failure of lower levels of self-control is challenged by studies showing that many patients cease drug use at an age when their lives entail more responsibilities (35, 39, 40). It also does not fit with findings that people suffering from SUDs were able to choose money over their drug of preference in experimental settings (41), and as part of treatment (42).

Snoek (34) views SUDs as mainly a disturbance of a higher level of self-control that overlaps closely with our notion of authenticity in that dependence, abuse and craving interfere with the person's ability to act in accordance with their values. Lower levels of self-control like impulsivity often play a role in SUDs, but they are rarely the whole story.

Autonomy is affected by SUDs in many different ways that involve both competence and authenticity. For example, it can change the way patients view themselves. The long-term impact SUDs have on people's bodies (e.g., energy levels, illness, risk of overdosing when relapsing, the reaction of others to their appearance), as well as on their self-esteem, can lead them to stop setting goals for themselves and lose belief in their own competence. People living with SUDs are disproportionally vulnerable to social adversity such as poverty, unemployment, and homelessness, which often forces them to abandon plans (43). They often come to believe they are not able to live the life they value, or be the kind of person they value. They may start to believe that living with a SUD is the life that is meant for them, identifying themselves with the lifestyle of a person with a SUD. Social interactions might reinforce such beliefs, providing them with experiences that negatively impact on self-esteem, such as repeatedly being rejected in job interviews. They may give up on the pursuit of other goals that do not relate to their addictive lifestyle because of this loss of self-belief (43). This in turn can impact upon authenticity: their actions, driven by short term satisfaction, may no longer make sense to them given their other desires and values. The patient may find themselves moved by desires and values they do not endorse, making it more difficult to have an integrated sense of who they are and what they want in life.

In sum, in SUDs there is an interplay in how patients are affected in their competence and authenticity. Social adversity, as well as the psychological and physical costs of SUDs, may lower the patient's self-belief and ability for self-control, making it more difficult to pursue the values and desires they endorse or to make plans in the first place. People living with SUDs often come to believe they are not able to live the life they value and give up on the pursuit of goals that they take to be unattainable because they believe they are unable to make a difference to the life they lead, much like the learned helplessness seen in depression.

#### Autonomy in Obsessive Compulsive Disorder

Obsessive Compulsive Disorder (OCD) is characterized by obsessions (recurrent and persistent thoughts or images) and/or compulsions (repetitive ritualistic behavior or mental activities) (26). Obsessions are defined by recurrent and persistent thoughts, urges or images that are experienced, at some time during the disturbance, as intrusive, unwanted, and that in most individuals cause marked anxiety or distress. The individual attempts to ignore or suppress such thoughts, urges, or images, or to neutralize them by performing a compulsive mental or physical action. Compulsions are defined as repetitive behaviors (e.g., hand washing, ordering checking) or mental acts (e.g., praying, counting, repeating words silently) that the person feels driven to perform in response to an obsession, and according to rules that must be applied rigidly. The behaviors or mental acts are aimed at preventing or reducing distress or preventing some dreaded event or situation. However, these behaviors or mental acts either are not connected in a realistic way with what they are designed to neutralize or prevent, or are clearly excessive (26).

Most OCD patients recognize their fears are excessive or irrational. Nevertheless, this insight does not stop them from performing compulsions. They fail in attempts to keep control over their obsessions, and feel compelled to perform compulsive behavior to neutralize anxiety and distress. Consequently, OCD patients feel unfree because they are unable to do the things they want to do. The compulsions take up a lot of time and energy of the patient, which leads to an increasingly small world because of the lack of time to normally participate in daily life. Patients are motivated by immediate irresistible urges or overwhelming aversions, that may compete with the conception of what they want their lives to look like when viewed from a long-term perspective, such as the pursuit of a career or taking care of their family (44).

This conflict between the compulsive behavior and long-term goals causes internal struggle. One might argue that this internal struggle is a loss of self-control which is felt very acutely by patients and diminishes their self-efficacy and self-esteem. This might negatively affect their competence just as was argued to be the case in addiction.

Paradoxically, patients with OCD try to regain control over their lives by deliberation and paying extra attention to their behavior, which in fact has the effect of diminishing, instead of increasing, their sense of agency (45). De Haan et al. (45) describe a process of hyper-reflection in OCD patients which starts with a feeling of insecurity, anxiety or tension. This leads to attempts to regain control through deliberation and reflective action (e.g., trying to consciously control the performance of one's actions). This analyzing and paying attention to actions may lead to an increase in insecurity, which again leads to anxiety and tension.

Most OCD patients are not alienated from the beliefs and values that motivated their actions before the onset of their illness. Whereas, some patients with MDD or SUDs may cope with the internal conflict between values and behavior by abandoning plans, in general most OCD patients realize throughout the course of the disorder that their compulsions are nonsensical, and incompatible with their goals and values in life. Most OCD patients can distinguish between what they feel compelled to do by their OCD symptoms and how they would like to spend their time and energy. However, there are also cases where the severity of the OCD symptoms makes patients doubt about themselves, and what they might be willing to do. For example, a patient with severe OCD with focus on sexual obsessions about little children might start to doubt herself and become confused about whether she has indeed harmed a child. She might begin to question a core belief about herself that she would never harm children. Here we observe that the severity of OCD begins to interfere with her sense of who she is and what she wants, affecting her authenticity.

In sum, we argue that competence is disturbed in OCD. The person's anxiety has the consequence that they are sometimes unable to act effectively on their intentions, and instead feel strongly compelled to engage in ritualistic behaviors that aim to reduce anxiety. Paradoxically, attempts to regain competence by reflection and deliberation may actually lead to more anxiety and feeling of loss of control. Furthermore, more severe OCD symptoms can cause a patient to question their own moral resolve in the light of their obsessions and compulsions, making them confused about their identity, and disrupting their authenticity.

#### Autonomy in Anorexia Nervosa

Anorexia Nervosa (AN) is a life- threatening mental disorder that is characterized by compulsive starvation and other weight loss behaviors, often resulting in malnutrition, severe emaciation and in the most severe cases, death (46). The main criteria are restriction of energy intake relative to energy requirements, resulting in a significant low body weight relative to the age, sex, developmental trajectory, and physical health of the individual (less than minimally normal/expected). The intense fear of gaining weight or becoming fat leads to persistent behavior that interferes with weight gain. This behavior can persist even when there is a significant low body weight, there is a disturbed perception of body weight or shape. The patient's self-worth is also disproportionately influenced by the perception of body weight or shape, and by persistent lack of recognition of the seriousness of low bodyweight (26).

In our clinical work at the Amsterdam UMC we have observed that patients suffering from severe AN struggle with what they want for themselves and what they want at times of severe AN, affecting their sense of self and their self-esteem. In interviews conducted with anorexic patients, Hope and colleagues (47) confirm this observation, describing AN as a struggle, in which patients were not content with their “life choice” of anorexia. For example, a patient who does not want to gain weight and limits her food intake often shows remarkable levels of constraint in her choices, as well as the ability to attain goals she sets for herself. However, this extreme self-control may also interfere with the pursuit of other long-term goals such as career or study goals due to lack of concentration, and the physical problems associated with low weight.

Many of the patients in the study of Hope and colleagues report viewing AN as separate from themselves (47, 48). This separation was described in different ways. For example, some patients articulated the idea of two parts of themselves – what Hope and colleagues call an “authentic self” and an “inauthentic self.” It was common for patients to describe a power struggle between these different parts of themselves. A patient in our hospital described this struggle as “confusing and saddening”, she knew rationally it would be better to postpone or put-off purging but she also felt compelled to continue this pattern of behavior. Hope et al. (48) argue that affective components, particularly fear and anxiety, can dominate beliefs in such a way that the anorexic's grounds for making a decision (to purge for example) may differ from the reasons they give to justify this decision. These emotions of fear and anxiety can interfere with the patient's competence insofar as they can lose the ability to recognize which preferences and beliefs they endorse as expressing who they are.

This confusion notwithstanding, many participants describe AN as integral to who they are, as a part of their identity. One patient described AN as something she has become: “it defines who you are, as opposed to just an illness that you have” (47). This integration of AN into the person's identity may also undermine their ability to decide on treatment interventions that require them to put on weight (48). Sato (49) suggests that anorexic patients are almost always trapped in a dilemma in which they are frightened of change brought about by treatment. On the one hand they fear losing their sense of being in control, while on the other hand, they are distressed about their physical deterioration, as a consequence of losing weight.

The ability and inclination to exercise excessive conscious control over their behavior is part of AN that results in diminished autonomy, not unlike OCD patients. Patients cannot get out of the dilemma of losing their sense of control and being distressed about their physical deterioration on their own. Yet they are also frequently too frightened to seek help (49). This dilemma and the high morbidity rate seen in AN, makes the question of how much autonomy is disturbed by this disorder even more critical than the other disorders we have discussed.

Using our analysis of autonomy, competence might in some respects be thought to be intact in AN. The person with AN can for instance competently forms goals and intentions and act on them. Whether the person is able to critically reflect on their values and desires is however debatable, thus impairing their authenticity. The more severe AN, the more doubt there might about how whether the behavior of the person with AN fits with who they want to be. The anorexic's interpretation of who she is and where she wants to go in life in the longer term, might not fit with obsessively losing weight (Recall our discussion of the importance of taking a long-term perspective on patients in section **The Authenticity Condition**). There might be some degree of ambivalence, but also a certain amount of self-deception which we will see is also common in psychosis, our final example.

#### Autonomy in Schizophrenia

Schizophrenia is characterized by two or more of the following symptoms: delusions, hallucinations, disorganized speech, grossly disorganized or catatonic behavior and/or negative symptoms (e.g., diminished emotional expression or avolition) (26). Patients diagnosed with schizophrenia can thus experience positive symptoms (such as delusions, hallucinations) concurrent with negative symptoms (loss of volition, withdrawal, muteness).

Autonomy in schizophrenia can be compromised in different ways. Symptoms like delusions and hallucinations often lead to fear, and a disconnect between patients and their environment in how they experience the world around them. This can lead to social withdrawal and the inability or unwillingness of the patient to engage in their daily activities such as work, study or family life. While it is possible that patients may experience an increase of autonomy during psychosis, for instance by having grandiose ideas about themselves, for most patients these psychotic periods are temporary and transitory. Sass (50) argues that it is common for patients suffering from delusions to experience exaggerated autonomy. He questions how accurate it is to characterize schizophrenia as being a condition of diminished autonomy, or agency. He states that schizophrenia patients can report, alongside experiences of passivity, also experiences of abnormal degrees of activity or control.

Following a period of psychosis, patients often struggle with how their sense of reality has shifted, and often feel ashamed or distant from the behavior and ideas they had during their psychosis. By changing the patient's experience of reality so rapidly, psychotic symptoms diminish the patient's autonomy at a very basic level, compromising their decision-making capacity, their sense of self and relation with the world around them.

The negative symptoms such as loss of volition, withdrawal, inactivity and muteness that can be present in schizophrenia lead to disturbances of autonomy comparable to those seen in major depression (MDD). Negative symptoms are characterized by a decrease in activity, difficulty in maintaining a daily routine, and loss of interest and engagement in activities. Social withdrawal, loss of volition and inactivity have the consequence that the patient feels unable to significantly affect the direction of their lives through their own actions. With the loss of volition their ability to make their plans and intentions effective is compromised. Furthermore, the lack of interest and loss of volition can impact on the person's motivation to act on their beliefs and values. The person with schizophrenia might still have beliefs about how they want to live, but be unable to get themselves to act upon these beliefs (again similar to what we described in patients with major depression).

Finally, cognitive impairment is a core feature of schizophrenia that additionally impacts autonomy. Cognitive impairment in schizophrenia involves a broad array of non-social and social cognitive domains, and is strongly associated with functional impairment (51, 52). Non-social cognitive domains that are commonly impaired are speed of processing, working memory, attention/vigilance, verbal learning and memory, visuospatial learning and memory, and reasoning and problem solving. Compromise in these domains can diminish autonomy on a very basic level: for instance problems with memory, planning and organization may hamper daily activities including the ability to work, which in turn may affect the patient's ability to live independently (52). Indeed, these neurocognitive impairments have been shown to be strongly affecting real life functioning in people with schizophrenia (53).

Studies investigating social cognition in schizophrenia found impairments in emotional processing/perception and mentalizing. A smaller number of studies have investigated social perception and attributional bias, where they found impairments in social perception (ability to identify social roles, social rules and social contexts from non-verbal cues) (52). These deficits in social cognition can affect the person's ability to navigate social life and thereby affect their ability to lead the life they desire.

In sum, disturbances of autonomy specific to schizophrenia include how the patient's perception of reality can change during psychosis, impacting on their decision-making capacity. Some patients experience loss of motivation comparable to that seen in MDD. Finally, people with schizophrenia experience a broad range of deficits in (social) cognitive functions. Together, these factors limit the patient's autonomy in a very fundamental way, making it difficult for the patient to navigate the world, let alone make decisions that lead to them living a fulfilling life.

### Stigma and Autonomy

Another factor that might negatively impact autonomy is stigma, which is common to all mental illnesses. Stigma is defined as the co-occurrence of the components of labeling, stereotyping, separation, status loss, and discrimination (54). Self-stigma (i.e., internalized stigma) is associated a range of autonomy relevant outcomes such as self-esteem, impaired social relationships, increased suicide risk, poorer vocational functioning, avoidant coping an decreased service engagement (55, 56). Indeed studies showed a link between self-stigma and empowerment self-esteem, self-efficacy in people with schizophrenia (56, 57) who are at high risk of suffering from stigma and self-stigma (56). Stigma in people with substance use disorder can be more complicated because they are often blamed for their conditions. Research showed that people with alcohol dependence provoke more social rejection, negative emotions, are at risk of structural discrimination, and may not always receive optimal healthcare through negative attitudes among health care professionals (58). Moreover, internalization of stigma can lead to a loss of self-respect, decreased self-esteem and loss of self-efficacy, decreasing their autonomy (as described in the section Autonomy in Substance Use Disord on SUDS) and decreases their changes of recovery (59, 60).

In sum (self)- stigma, can deprive a person of the ability to act effectively on their intentions, engage in treatment successfully or receive optimal healthcare, let alone pursue their goals, compromising autonomy through both competence and authenticity.

### Discussion and Conclusion

Our discussion of the disturbance of autonomy in five mental disorders has allowed us to identify how competency and authenticity can be compromised in specific ways by mental illness. Recall that competence refers to the ability to carry out plans and act in accordance with one's goals, intentions and preferences. Authenticity refers to how the self relates to their beliefs, desires, commitments and values that motivate them to act. In order to be autonomous a person must not only be able to make their fundamental motivations effective in how they act. In addition, the desires and values they act upon must be such that they would endorse them were they to reflect on the matter. If they were to act on desires and motivations they did not accept or endorse, this would undermine the authority they have over their actions. In short, they would fail to be self-governing.

We have attempted to identify differences and overlap in how competence and authenticity were disrupted in five different mental disorders. However, before empirical investigation of these ideas will prove possible we need to identify measures of autonomy that could, for instance, be included in a scale for evaluating disturbances of autonomy. In the first part of this discussion we will attempt to identify patterns of disturbance across the five mental disorders that could potentially serve as the basis for such measures. In the second part, we sketch some ideas for how autonomy could be specifically targeted in psychotherapy.

#### Patterns of Disturbance of Autonomy in Mental Illness

In MDD and addiction both competence and authenticity are affected. In both disorders the loss of belief in their own competence is a central feature that may lead to the abandonment of plans and may disrupt the patient's sense of who they are and what they value (authenticity). Like in addiction and MDD, OCD patients also experience a loss of competence: the patient's ability to act effectively on their intentions that are in accordance with their values is disturbed by the urge to perform compulsions. They also experience a loss of authenticity in that patients act on motivational states they do not endorse (i.e., compulsions in response to anxious or uncomfortable feelings). Yet it is rarer in OCD that this leads patients to abandon plans and values that affect their sense of identity. By contrast, in AN the desire to lose weight is often more integral to the sense of who they are which can more directly affect their sense of identity and thereby authenticity. In schizophrenia, autonomy can be affected by the negative symptoms in a similar way as MDD, yet during psychosis competence is affected in an even more fundamental way where a rapid change in sense of reality compromises decision making, and hereby autonomy.

Moreover, we believe that authenticity is more often compromised by affecting patients sense of identity in more severely affected patients. For example, in severe OCD the struggle between a person's values, and compulsive behavior can ultimately lead to ambivalence in one's identity. Patients may doubt about how much of their behavior fits with their understanding of who they are. In MDD, feelings of worthlessness and doubts about self-efficacy can significantly affect the patient's identity: they may come to believe they truly are a worthless person. Likewise, addicts often come to believe they are not able to live the life they value, giving up on the pursuit of goals they value because they believe they are not able to achieve them.

The patterns we identified in the different disorders are generalization, but it is important to realize that how autonomy is affected differs between individuals, over time and between different contexts. How mental illness affects autonomy depends on the interplay between the particular individual and their (social) environment, their perspective on mental health issues and broader ideas about what is important in life, as well as their history and moment in time. For instance, while many patients with addiction may suffer from feelings of demoralization at certain times in their lives, such feelings may be more profound in circumstances where patients lack social support or any means to actualize their goals. Moreover, certain individuals through their perspective on life, their supporting social environment, or a combination of both, may be better able to find ways to live a fulfilling life within the limits of their symptoms. This is also something that can change or develop over time within individuals.

#### Clinical Implications

Qualitative and autobiographical research on recovery and severe mental illness found personal agency and autonomy to be important aspects in the patient's self-management of their mental illness. In this research, participation in valued activities and social roles have been found to be of importance in recovery and are therefore suggested to be integrated within medical services (61). These findings support the important role of autonomy in the maintenance and recovery of mental disorders. The concept of recovery in mental health care is defined as a personal process of living with mental illness. A useful review has identified five dimensions relevant to recovery, consisting of connectedness, hope and optimism, identity, meaning in life, and empowerment, and collectively abbreviated as CHIME (62–64). These five dimensions of personal recovery overlap in interesting ways with autonomy. Roe and colleagues (64) go so far as to identify “subjective” or “personal recovery with reclaiming autonomy or self-determination regardless of whether one does or does not clinically recover from illness” (p.133). For example, identity and meaning in life might strongly be associated with authenticity, referring to how the self relates to the beliefs, desires, commitments and values that motivate a person to act. Knowledge about and reflection on one's beliefs, desires or values, might strengthen one's identity and meaning in life and vice versa. Empowerment relates to competence, the person's ability to form goals and intentions and to act effectively on them. A person experiences empowerment to the extent that they succeed in acting effectively upon their valued goals and intentions, an ability associated with what we have called competence.

Studies in schizophrenia related disorders show no or partial correlations between clinical and personal recovery suggesting that personal recovery (and by extension autonomy) are related but distinct phenomena from what is currently included in clinical measures of recovery (63, 64). Emphasizing the importance of taking personal recovery or autonomy into account in treatment.

Treatment interventions that target autonomy (and by extension personal recovery) more directly, and hereby possibly affecting aspects of personal recovery rather than clinical recovery, may provide a different avenue to decrease suffering in mental illness. This avenue would focus on the possibility of the patient to live a meaningful and fulfilling life even in the midst of (severe) mental illness (a core idea behind personal recovery) (62). Most used current clinical measures focus mainly on symptom reduction and level of functioning (62). We have seen above how mental illness such as SUD and MDD can cause patients to no longer be moved by their goals and values. This can happen when patients become preoccupied with the suffering that accompanies their illness, when the illness becomes deeply ingrained in their identity or through self-stigma (as discussed in section Stigma and Autonomy). Interventions that are focused more directly on strengthening autonomy could be aimed at regaining purpose and meaning in life by reorienting the patient's thoughts to target their needs, values and goals. To do this, it is important to take into account the interaction between competence and authenticity. Thinking about how one wants to live and the person one wants to be does not by itself necessarily increase autonomy. The person also needs to be able to effectively act on their goals and intentions they set for themselves and believe in their own efficacy. This is consistent with the CHIME model of personal recovery which contains meaning in life and empowerment as important elements of personal recovery (62). Whereas personal recovery frameworks emphasize psychosocial interventions to increase autonomy and recovery, we believe it is important to also target more underlying psychological constructs such as self-esteem, self-efficacy, sense of identity with psychotherapeutic interventions. This way, psychosocial and psychotherapeutic interventions complement each other. Clinical interventions that aim to cultivate personal autonomy in patients should additionally aim at helping people to make choices and to set and achieve goals that align with their values. This may help to strengthen the person's self-efficacy and self-worth. This proposed direction is more directly aimed at targeting autonomy than other psychotherapeutic orientations, such as cognitive behavioral therapy (CBT).

For further research we believe it is important to empirically investigate these ideas about the relationship between autonomy and clinical treatment. For this we will need validated instruments to measure autonomy in psychiatry, for instance using interviews or questionnaires. Moreover, these instruments could be used to evaluate the effects of interventions aimed to strengthen autonomy in psychiatry, either as stand-alone treatments or as a part of existing therapies. Finally, we believe it is important to get a better understanding about the relationship between autonomy, quality of life and self-esteem in psychiatric disorders. We have been arguing that the positive effect that autonomy may have on these life domains may alleviate suffering in psychiatric patients.

## Conclusion

In this paper we examined the currently poorly understood, relationship between autonomy and mental illness. We have seen that there are many ways in which autonomy can be disturbed by mental illness. We have distinguished between the dimensions of competence and authenticity as disrupted in general by mental illness. We have described general patterns of how autonomy can be differently affected in five prototypical mental disorders. The effects on autonomy are expressed differently according to the underlying psychopathology. Moreover, there will inevitably also be individual differences in how each person will be affected in their autonomy, depending on their specific personal history, outlook on life, and their social context. We must take the wider environmental context of the individual, which may also change over time, into account when considering the effects of mental illness on autonomy within each person. Finally, we have suggested that psychotherapeutic interventions should target autonomy by restoring authenticity and strengthening competence. In doing so the person may be able to find ways to live a meaningful and fulfilling life even if their symptoms do not improve by more conventional clinical measures.

## Author Contributions

JB, JL, and JK carried out literature research, wrote the manuscript, and drafted the manuscript. DD and CB helped to draft the manuscript. All authors contributed to the article and approved the submitted version.

## Conflict of Interest

The authors declare that the research was conducted in the absence of any commercial or financial relationships that could be construed as a potential conflict of interest.

## Publisher's Note

All claims expressed in this article are solely those of the authors and do not necessarily represent those of their affiliated organizations, or those of the publisher, the editors and the reviewers. Any product that may be evaluated in this article, or claim that may be made by its manufacturer, is not guaranteed or endorsed by the publisher.
